# Increased Cerebrospinal Fluid Biomarkers of Neurodegeneration in Acquired Progressive Ataxia and Palatal Tremor Following a Static Lesion: A Case Report

**DOI:** 10.1002/mdc3.14247

**Published:** 2024-10-25

**Authors:** Carlo Fazio, Simone Regalbuto, Sebastiano Arceri, Davide Comolli, Alessandra Calculli, Piergiorgio Grillo, Giuseppe Cosentino, Liliana Brambilla, Daniela Rossi, Antonio Pisani

**Affiliations:** ^1^ Department of Brain and Behavioral Sciences University of Pavia Pavia Italy; ^2^ IRCCS Mondino Foundation Pavia Italy; ^3^ Marlene and Paolo Fresco Institute for Parkinson's and Movement Disorders, NYU Langone Health New York USA; ^4^ Istituti Clinici Scientifici Maugeri IRCCS Pavia Italy

**Keywords:** ataxia, palatal tremor, PAPT, secondary neurodegeneration, tau

Progressive ataxia and palatal tremor (PAPT) is a rare syndrome combining palatal tremor and progressive cerebellar ataxia. PAPT is classified into familial and sporadic forms, but etiopathogenesis remains largely unclear.^1^ Recent neuropathological and positron emission tomography studies^2^, ^3^ revealed tau deposition in brains of patients affected by sporadic PAPT, suggesting it may be a novel tauopathy.

Another condition, termed “oculo‐palatal tremor and tardive ataxia”^4^ has been described as a rare complication of large brainstem lesions with onset from months to years following a monophasic lesion.^4^, ^5^ It has been suggested that this syndrome could be considered as an acquired or structural form of PAPT (sPAPT).^1^ Although the progressive course of sPAPT suggests a neurodegenerative phenomenon,^4^, ^5^ to date no clear evidence in support of this hypothesis has been presented.

## Case Report

A 70‐year‐old male presented with progressive gait ataxia, dysarthria, and oscillopsia over the past 3.5 years. Past medical history, included mild hypertension and atrial fibrillation treated with amiodarone. Four years before he experienced a spontaneous right pontine‐midbrain hemorrhage treated with surgical drainage. Post‐surgery, his neurological exam showed mild left pyramidal signs, vertical and horizontal gaze palsy, mild imbalance, and upper right limb ataxia. After rehabilitation, he reported full recovery of gait issues.

However, 8 months later, he began to develop progressive imbalance, dysarthria, and oscillopsia. A magnetic resonance imaging (MRI) showed hemosiderin deposition in the right pontine‐midbrain and T2‐weighted hyperintensity in the inferior olives, indicating hypertrophic olivary degeneration (HOD), a hallmark of PAPT. A DaTscan ruled out presynaptic dopaminergic denervation. Two years later, he developed dyspnea and throat tingling. An otorhinolaryngologist observed rhythmic rest laryngeal spasms and referred the patient to our clinic. Neurological examination on admission revealed stable signs because of previous hemorrhage, including left sensory‐motor deficit, upper right limb ataxia, vertical gaze supranuclear palsy, bilateral restriction in abduction of both eyes (more severe on left), and left hypotropia (Video 1). Additionally, he showed a progressive cerebellar syndrome with prominent trunk, gait, and speech ataxia. He also exhibited palato‐laryngeal rhythmic activation consistent with palatal tremor and a synchronous pendular nystagmus in all directions of gaze, more evident in left eye (Video 2).

**Video 1:** Extraocular movements: pendular nystagmus, vertical sopranuclear gaze limitation, bilateral restriction in abduction of both eyes (more severe on left), skew deviation with left hypotropia, saccadic pursuit.

**Video 2:** Oculo‐palatal tremor.

**FIG. 1:**
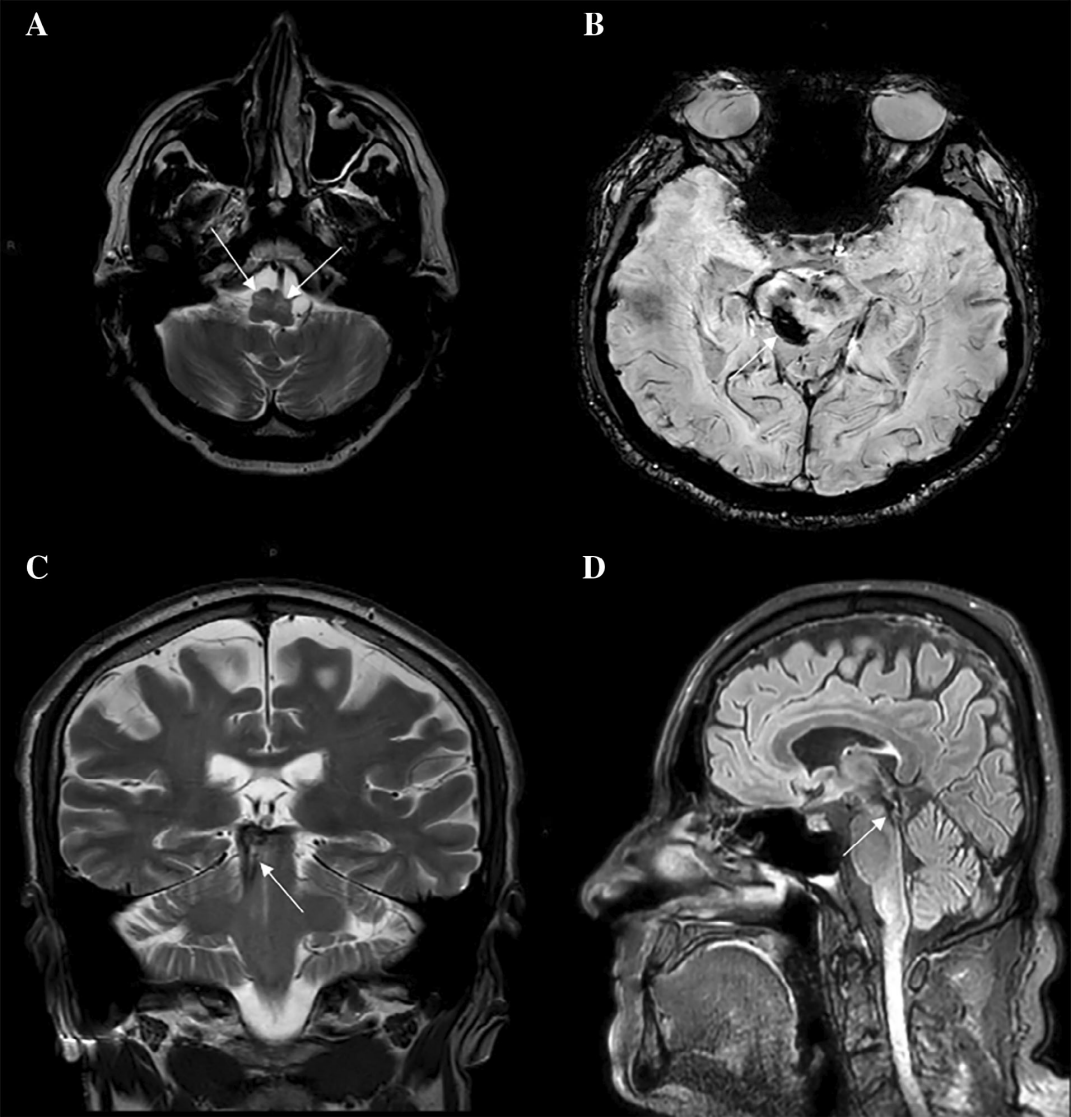
Magnetic resonance imaging findings. (**A**) Axial T2‐weighted bilateral hypertrophic olivary degeneration (arrows). (**B**,**C**) Susceptibility weighted imaging sequence and coronal T2‐weighted showed hemosiderin accumulation in right midbrain, superior cerebellar peduncle and pons. (**D**) Sagittal fluid attenuated inversion recovery (FLAIR) showed clear involvement of Guillan‐Mollaret triangle but no signs of cerebellar atrophy.

**FIG. 2:**
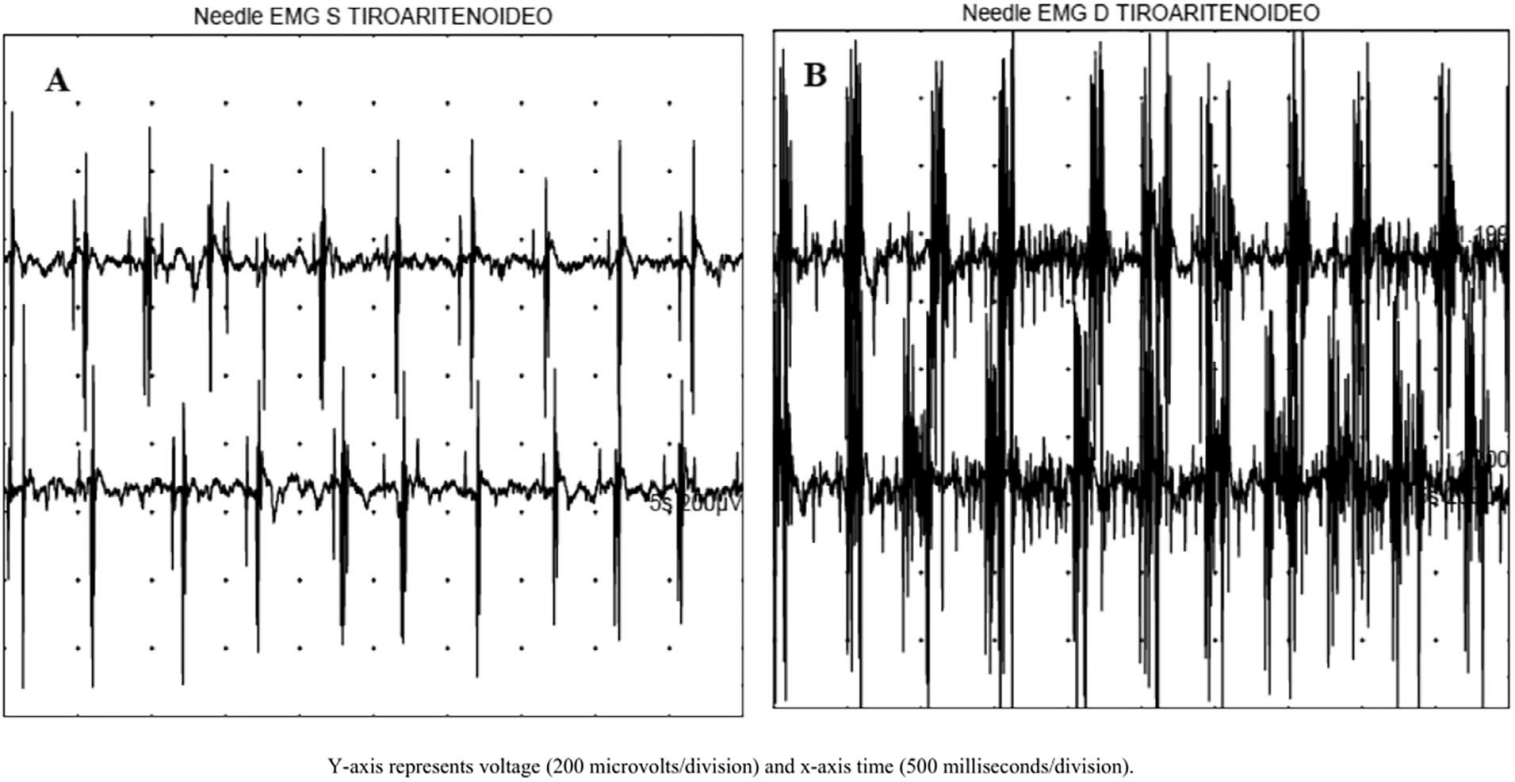
Concentric needle electromyography (EMG) study of laryngeal muscles. EMG traces show pseudo‐rhythmic activation frequency of ~2‐Hz in left and right thyroarytenoid muscle (**A**,**B**). Bursts duration ranges from about 10 to 200 ms suggesting a laryngeal myoclonus.

MRI confirmed bilateral HOD and persistent hemosiderin deposit affecting midbrain, pons, and superior cerebellar peduncle, without significant cerebellar atrophy (Fig. 1). Electromyography (EMG) of laryngeal muscles showed a 2 Hz pseudo‐rhythmic activation of thyroarytenoid and cricoarytenoid muscles, indicating larynx involvement (Fig. 2). A genetic screening for ataxia covering nearly 50 genes (next generation sequencing panel) ruled out pathogenic mutations associated with spinocerebellar ataxia (SCA) as well as familial PAPT forms (*POLG*, *GFAP*, and SPG7). SCA20 and neurodegeneration with brain iron accumulation 3 (NBIA3), instead, were considered improbable based on neuroimaging. Neuropsychological tests indicated mild deficits in frontal‐executive functions. Cerebrospinal fluid (CSF) analysis (chemiluminescence enzyme immunoassay) revealed increased levels of total tau (t‐tau) (844 pg/mL, normal values <404 pg/mL) and phosphorylated tau (p‐tau) (113.60 pg/mL, normal values <56.5 pg/mL) proteins, whereas β‐amyloid levels were normal. Neurofilament light chains (Nf‐L) were also elevated (1.12 ng/mL) (NF‐light ELISA CE). The patient was discharged with gabapentin 900 mg/daily. After 6 months, there was slight improvement in palatal tremor and oscillopsia, but speech impairment and ataxia remained unchanged.

## Discussion

sPAPT is a rare condition characterized by distinct clinical and neuroimaging features. Our patient demonstrated typical signs, including progressive cerebellar ataxia and oculo‐palatal tremor, which were supported by brain MRI findings and consistent EMG data. Our case provides novel EMG data on laryngeal involvement, which is rarely reported.

Most notably, our patient showed elevated levels of CSF t‐tau, p‐tau, and Nf‐L, indicating a potential neurodegenerative process. Other tauopathies such as Alzheimer's disease, chronic traumatic encephalopathy, progressive supranuclear palsy, and cortico‐basal degeneration were deemed improbable based on clinical presentation and negative auxiliary tests. Moreover, it was unlikely that the elevation of CSF biomarkers was a residue of the neuronal damage occurred during the acute cerebrovascular event. CSF tau levels, indeed, usually rise within hours, peak at 5 to 7 days, and normalize after few months following stroke.^6^ To our knowledge, this is the first report of elevated neurodegeneration CSF biomarkers in sPAPT. These findings, associated with progressive clinical course and onset of symptoms after static lesion, support a degenerative process not only in sporadic cases of PAPT, but also in structural forms, hence differentiating sPAPT from other forms of acquired PAPT (ie, infectious or autoimmune).

In our patient, temporal correlation with hemorrhagic stroke suggests a secondary rather than primary neurodegenerative phenomenon. As already suggested by other authors,^5^ persistent hemosiderin deposition, evident in our case, may play a crucial role, potentially triggering secondary neurodegeneration.

Ferroptosis, a type of iron‐dependent cell death, is being investigated as a potential mechanism in various neurodegenerative conditions.^7^ Iron can bind to tau, inducing its phosphorylation,^8^ and iron overload may lead to tau accumulation and hyperphosphorylation.^9^ In turn, tau and p‐tau accumulation might further disrupt iron homeostasis, creating a vicious cycle that leads to neuronal degeneration.^10^ Although secondary neurodegeneration in our case remains speculative, we propose that persistent hemosiderin and elevated tau and p‐tau levels in CSF could result from iron‐tau interaction.

## Author Roles

(1) Research project: A. Conception, B. Organization, C. Execution; (2) Statistical Analysis: A. Design, B. Execution, C. Review and Critique; (3)Manuscript Preparation: A. Writing of the First Draft, B. Review and Critique.

C.F.: 1A, 3A

S.R.: 1A, 3B

S.A.: 1C, 3B

D.C.: 3B

A.C.: 3B

P.G.: 3B

G.C.: 3B

L.B.: 1C

D.R.: 1C, 3B

AP: 1A, 1B, 3B

## Disclosures

**Ethical Compliance Statement:** The authors confirm that the approval of an institutional review board was not required for this work. Informed patient consent was regularly obtained for video usage. Both oral and written consent was obtained. We confirm that we have read the journal's position on issues involved in ethical publication and affirm that this work is consistent with those guidelines.

**Funding sources and Conflict of Interest:** The work is supported by NEXTGENERATIONEU (NGEU) and funded by the Ministry of University and Research (MUR), National Recovery, and Resilience Plan (NRRP), project MNESYS (PE0000006) – A Multiscale integrated approach to the study of the nervous system in health and disease (DN. 1553 11.10.2022), and by Italian Ministry of Health “Ricerca Corrente 2022–2024” granted to IRCCS Mondino Foundation. The authors declare that there are no conflicts of interest relevant to this work.

**Funding Disclosures for the Previous 12 Months:** The authors declare that there are no additional disclosures to report.

## Data Availability

The data that support the findings of this study are available from the corresponding author upon reasonable request.
